# Magnetospectroscopy of shallow donors in two dimensions in the presence of fluctuations of the electrostatic potential

**DOI:** 10.1515/nanoph-2023-0689

**Published:** 2024-01-19

**Authors:** Krzysztof Karpierz, Michał Szot, Tomasz Wojtowicz, Jerzy Łusakowski

**Affiliations:** Faculty of Physics, University of Warsaw, ul. L. Pasteura 5, 02-093 Warsaw, Poland; Polish Academy of Sciences, International Research Centre Mag Top, Institute of Physics, al. Lotników 32/42, 02-668 Warsaw, Poland; Polish Academy of Sciences, Institute of Physics, al. Lotników 32/42, 02-668 Warsaw, Poland

**Keywords:** far-infrared spectroscopy, quantum wells, shallow impurities, localization, fluctuations of electrostatic potential

## Abstract

Spectroscopy of shallow donors is a tool to test theoretical models and to reveal properties of semiconductors. In this work we consider intra-shallow impurity transitions by studying a CdTe/(Cd, Mg)Te structure grown by a molecular beam epitaxy in which both a CdTe quantum well and (Cd, Mg)Te barries are uniformly doped with iodine donors. Measurements of a photocurrent (PC) at the far-infrared were carried out at 4.2 K and magnetic fields *B* up to 7 T with the energy of photons originated from a molecular laser in the range 2.2 meV–12.8 meV. Spectra (a PC signal vs. *B*, at a constant energy of photons) show lines which position does not depend on the photon energy but shifts with the in-plane electric field. These dependencies, which do not follow a well-established picture of shallow donor magnetospectroscopy in quantum wells, are explained within a model which unifies the role of fluctuations of the electrostatic potential and a magnetic-field induced electron localization.

## 1 Introduction

One of the main sources of success and role of semiconductors in advanced technology is possibility to tune their properties with doping. Multiple modern methods of fabrication of semiconductor structures and the ways of incorporation of dopants lead to necessity of studying impurities in new environments.

The amount of work, theoretical and experimental, devoted to impurities in semiconductors is huge. Shallow impurities in environments restricted by confining potentials were theoretically considered already in 60-ties of the 20th century (see, e.g. [1], where a donor on a surface of a semiconductor was considered) even before such ideas could come true. Predictions were put forward concerning a dependence of the energy of a shallow impurity with its position in a quantum well [2], [3]. When two-dimensional semiconductor structures became a reality, ideas formulated and well-established for shallow impurities in bulk systems had to be confirmed or developed to take into account the role played by a confining potential. Experimental studies comprised, in particular, spectroscopy in the far-infrared which – from the very beginning of application of this method [4] – concentrated on a dependence of intraimpurity transitions in the magnetic field. In quantum wells and heterostructures, doping with shallow impurities plays a decisive role in device engineering since the profile of dopants’ distribution is one of the main factors determining concentration of free carriers. A dependence of the spectrum of intra-impurity transitions on the position of a donor in the quantum well as well as properties of negatively charged donors (D^−^ centers) were determined in a number of papers [5]–[7].

Confinement of single impurities in quantum dots is another area of modern solid-state physics which requires both a technological perfection and advanced experimental techniques. Nevertheless, it was possible, for example, to demonstrate experimentally an optical orientation of a single Mn^2+^ ion [8] or a zero-field splitting for a single Co^2+^ ion in a quantum dot [9] (see also [10], [11]).

Technological and experimental developments have been accompanied with a theoretical support which aimed to explain observations [12], [13] or predict new dependencies. In particular, after resolving a general problem of a shallow impurity in a two – dimensional system [2], the main task which has been of importance and significance even nowadays is the modification of the structure of energy levels by external electric and magnetic fields, and pressure.

The electric field changes the energy of levels of bound electrons (or holes) which is called the Stark effect. Unfortunately, these changes are rather small due to small values of electric fields attainable in semiconductors and typically can be observed as a broadening rather than splitting of spectral lines [14]–[16]. A method to study the Stark effect on a shallow acceptor in a GaAs/(Al, Ga)As heterojunction by considering a luminescence between an electron in a two-dimensional electron gas and a hole localized on the acceptor was proposed in [17]. Generally, studying a near-band gap luminescence gives information about structure of shallow impurity levels as was evidenced, for example, in the case of GaAs in [18] or of GaN in [19]. In particular, these studies involve observation of pair spectra giving information about electrostatic interaction between charged impurities which can be directly translated into the strength of the internal electric field.

The evolution of the levels of an impurity with magnetic field, i.e., the Zeeman effect, gives a much stronger effects than the Stark effect does. Shifts of the energy of the levels in magnetic fields of a few T are of the order of meV which can be easily monitored by spectroscopic methods. Shallow donors in CdTe, which is the material of interest in this paper, have been studied in magnetic fields in a number of papers, e. g., in [20]–[23] to name a few.

A theoretical approach to describe the evolution of impurity levels in magnetic field is very often coupled with simultaneously considered influence of electric field. This approach can be found in the case of quantum wells (see. e.g. [12], [24]), wires (see, e.g., [25]) or dots (see, e.g., [26]). However, in all cases found during the literature research the electric field was directed in the direction of the magnetic field and the fields were directed perpendicular to the quantum well surface or along a symmetry axis of a cylindrical quantum dot. A crossed configuration was considered in [27] but in this case the magnetic field was directed in-plane. The experimental configuration of the present work directs the magnetic field perpendicularly and the electric field – in parallel to the quantum well. We will discuss this configuration in the Section 4.

A specific role which can be attributed to shallow impurities is treating them as probes of local properties of a material. In particular, a far-infrared spectroscopy of shallow donors allowed revealing a fluctuating pattern of the electrostatic potential resulting from a random distribution of charged centers in semi-insulating GaAs [28] and CdTe [23]. In the case of the former material, it was also possible to reveal a spacial correlation between positions of acceptors and the main structural defect, EL2 [29]. A comparison of this result with magnetotransport data allowed to experimentally show that the influence of local fluctuating electrostatic fields on material properties depends in a decisive way on the spatial extent of the electron wavefunction which serves as a probe in given experiment [30]. Specifically, if a donor wave function is the probe (in a spectroscopic experiment on intra-donor transitions), then it is strongly affected by fields resulting from charged acceptor-charged EL2 dipole and reveals the material as strongly disordered. On the other hand, in a transport experiment, it is the wave function of a conduction band electron which serves as a probe, it averages over these local fluctuations and the material seems to be of a high quality.

Energy separation of electron levels bound to a shallow impurity in a semiconductor falls within a meV range. For this reason, magnetospectroscopy in the far-infrared (FIR) is one of the basic methods to study shallow donors in solids [31], [32]. One of the most sensitive versions of this technique is based on measurements of a photocurrent (PC) resulting from interaction of FIR photons with a material studied. Such experiments are carried out by collecting Fourier spectra at subsequent values of *B* or by sweeping *B* when a monochromatic radiation is used. In this paper, we describe results obtained solely by the latter technique. In such a case, the magnetic field tunes the separation between levels of the shallow impurity and a resonant absorption occurs when this separation coincides with the energy of incident photons. Then, intra-impurity transition occurs and since ionization energy of excited states of a shallow donor is small, an excited impurity is thermally ionized and a PC signal is created (a magnetic field dependence of the PC signal is called a spectrum in this paper). In a PC experiment, a feature in the spectrum which corresponds to a resonant intra-impurity transition shifts in the magnetic field when the energy of incident photons is changed. A standard picture resulting from such spectroscopic measurements, confirmed by many experimental groups (see, e.g., [33]), shows a dependence of splitting of impurity levels on the magnetic field which can be further explained by an aprropriate theoretical model.

This paper reports experimental results which show a new aspect of intracenter donor transitions in quantum wells in the presence of the magnetic field. In PC spectra measured at the far-infrared on a single CdTe/(Cd, Mg)Te quantum well, uniformly doped with iodine donors in the well and in the barriers, a dominant peak, which is related to 1s–2p^+^ transition, keeps its position in the magnetic field irrespective of the energy of incident photons. On the other hand, the position of the peak in the magnetic field changes under application of the in-plane electric field. As one can notice, these observations do not fit into a standard picture of intra shallow donor transitions in magnetic and electric fields. To explain these results, we propose a model the main components of which are: the presence of fluctuations of the electrostatic potential, a continuous distribution of the ionization energy of shallow donors in the material studied and a magnetic-field induced electron localization.

The paper is organized as the following. Section 2 describes the experimental technique and results. The model and the interpretation of results is presented in Section 3 and in Section 4 we discuss and conclude the paper.

## 2 Experiment and results

A single quantum well (QW) structure was grown by molecular beam epitaxy on a 0.4 mm-thick semi-insulating (SI) (001) GaAs substrate. The layers’ stack involved a 7 μm-thick buffer of undoped CdTe, a 500 nm-thick Cd_0.8_Mg_0.2_Te barrier, a 16 nm-thick CdTe QW, and a 48 nm-thick Cd_0.8_Mg_0.2_Te barrier. The role of a thick undoped CdTe buffer is to increase the quality of the QW. In fact, as it was shown in [23], a mismatch of about 14 % between CdTe and GaAs lattice constants makes the CdTe/GaAs interface a region of a crystallographic disorder which propagates into the CdTe layer on the distance of about 4 μm. The buffer of undoped CdTe allows to decrease the density of dislocations down to about 10^7^ cm^−2^ in the QW. The Mg content in the barriers leads to the QW depth of 240 meV in the conduction band (CB). The first electronic level in the QW is situated at about 11 meV above the bottom of the CB. Both barriers and the QW were uniformly doped with iodine donors at the level less than 2 × 10^16^ cm^−3^, which is the concentration below the Mott transition [34] in CdTe. The Bohr radius of donor in bulk CdTe is $a_{B}^{*}$ = 6.4 nm and a mean distance between donors *d* corresponding to the doping level is *d* ≈ 37 nm, which is equal to about 5.8 $a_{B}^{*}$ . Therefore, the Mott criterion for isolated centres $d>4.5a_{B}^{*}$ is fulfilled. The binding energy of a shallow donor in bulk CdTe Ry* = 13 meV. Samples studied were cut to rectangular slabs with typical dimensions of 3 × 3 mm. Indium electrical contacts were prepared on the top surface by soldering.

The source of FIR radiation was a CO_2_-pumped molecular laser. We used photons with energies *hν* from 2.2 to 12.8 meV. The FIR radiation was guided to the sample by a stainless steel tube (an oversized waveguide). The sample was cooled with a helium exchange gas down to 4.2 K and shielded from a background 300 K radiation with a cold black polyethylene filter. At this temperature, the resistance of the sample exceeded 10^11^ Ω what made PC measurements practically impossible because of very long time constants of the system.

To deal with this problem, we applied an experimental technique that was successfully used in the past in the case of shallow donor magnetospectroscopy in SI – GaAs [28], [30] and CdTe epitaxial layers [23]. During measurements, the sample was continuously illuminated with a visible light (VIS). The energy of VIS photons was equal to 2.1 eV which was higher than CdTe QW band gap energy and lower than that of Cd_0.8_Mg_0.2_Te barrier. The VIS optical pump generates electrons in the conduction band and also increases population of neutral shallow donors. The latter effect occurs because an electron captured onto a donor center is localized and a recombination with the parent acceptor or valence band states is less probable. An increased population of neutral donors enables carrying out intra-donor spectroscopy experiments. Illumination of the sample with VIS light used during experiments causes the resistance drop to about 50 MΩ.

A very high resistance of samples cooled in the dark requires an additional remark. Such a resistance at 4.2 K could be expected because of a low doping level (donors are isolated and do not form a band), no room-temperature thermal excitation (shielding with a cold black polyethylene) and an energy of ionization of the donor ground state equal to 13 meV in comparison with a thermal energy of 0.35 meV at 4.2 K which means that only a very small fraction of the donors would be thermally ionized leading to a very high resistance in the absence of illumination. However, in samples of this kind there is another factor, i.e., compensation, which is introduced by acceptor states present on the surface of the sample. This was proved by magnetophotoluminescence experiments on similar samples grown in the same MBE machine at Institute of Physics, Warsaw, as samples analyzed in the present work. The basic experimental facts are presented in [35], [36] and the conclusion the most important for the current work is that acceptor states present on the surface of a (Cd, Mg)Te barrier (a cap layer) can dope the QW (positioned below the barrier) as effectively as doping with nitrogen acceptor does. What is more, the presence of a line in a photoluminescence spectrum attributed to recombination of an electron with a neutral acceptor in the QW was shown in [37]. A necessary condition to achieve a p-doping of the well with surface acceptors is to keep the thickness of the barrier small enough. As can be read from Figure 1 in [35], the thickness of the barrier of 48 nm in the sample studied in the present work is adequate for a p-doping leading to concentration of holes in the QW on the level of 10^11^ cm^−2^. Doping a 16 nm-thick QW on the level of 2 × 10^16^ cm^−3^ gives the donor concentration of 3.2 × 10^10^ cm^−2^ which means that one can expect that the QW is fully compensated.

**Figure 1:**
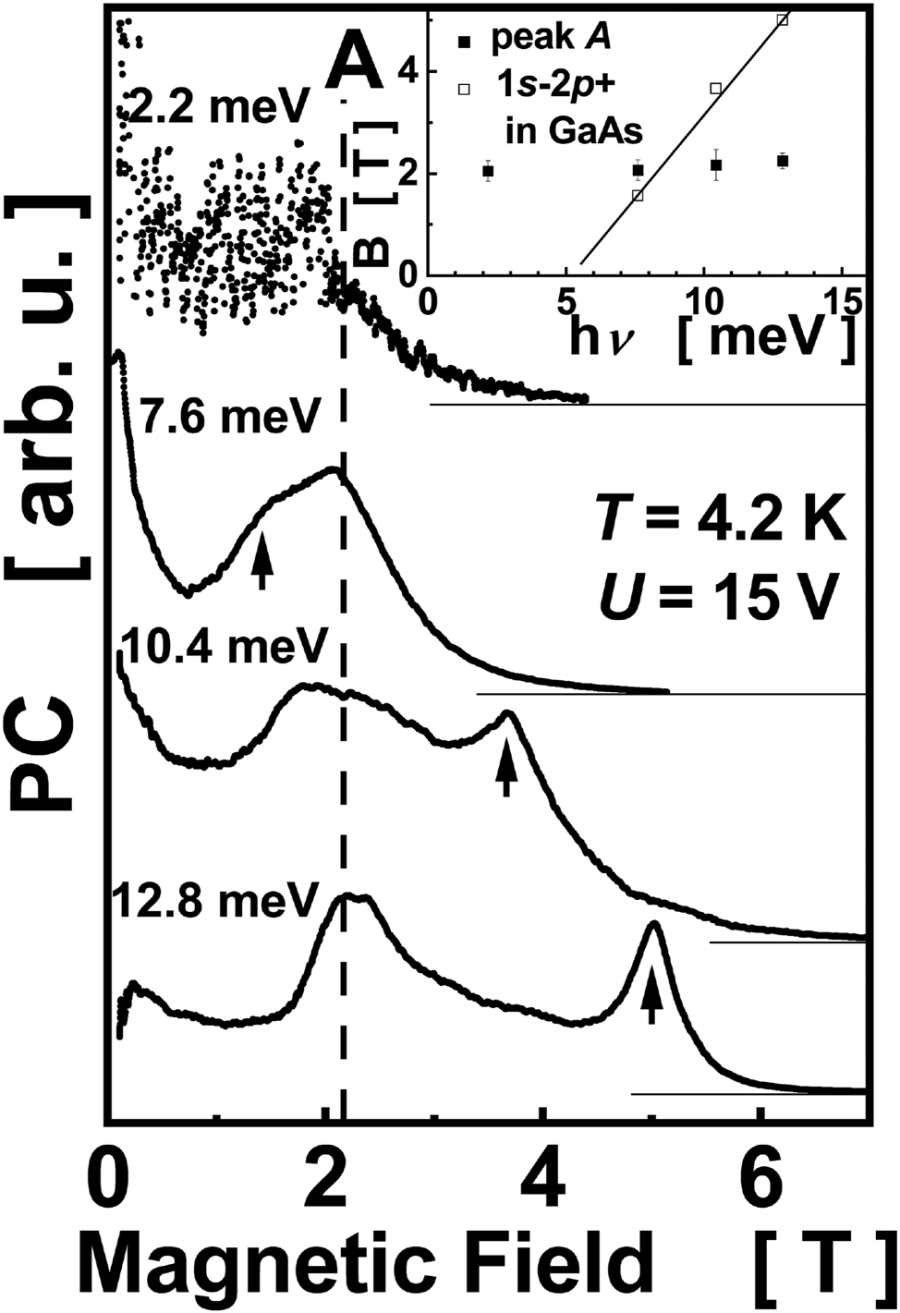
Evolution of PC spectra as a function of FIR photon energy at fixed bias voltage *U* = 15 V at *T* = 4.2 K. Vertical arrows mark 1s–2p^+^ transition in SI-GaAs. A dashed line – guide for the eye showing the position of peak A. Spectra are labeled with the energy of FIR photons and are shifted vertically for clarity. The inset shows the dependence of position of maxima in magnetic field *B* as a function of FIR photon energy *hν*. Uncertainities of the positions are marked. The solid line shows a *B*-dependence of 1s–2p^+^ transition in GaAs.

The sample was placed in the center of a superconducting coil and was biased with a voltage source in the range 10–30 V. The measurements were carried out at 4.2 K. The FIR laser beam was modulated with a mechanical chopper which allowed measuring the sample photoconductivity with a standard lock-in technique while the magnetic field, perpendicular to the QW plane, was swept between 0 and 7 T. The signal was measured as a voltage drop on a 1 MΩ load resistor connected in series to the sample.

Figure 1 shows spectra registered at a constant bias of 15 V at a few FIR photon energies *hν*. Each spectrum exhibits one or two peaks (regardless structures at low *B* which are treated as a background signal and will not be discussed in this paper). One of them, marked with vertical arrows, moves towards higher *B* with increasing *hν*. The position of this peak agrees very well with that of 1s–2p^+^ intra-shallow donor transition in GaAs and originates from donors in the SI-GaAs substrate. A possibility to observe a PC signal related to donors in GaAs substrate of CdTe quantum structures was previously reported in [23]; the signal appears due to a metastable population of donors resulting from illumination of the sample with a visible light. A detailed description of a shallow donor spectroscopy in SI-GaAs at liquid helium temperatures can be found in [28], [29]. The half-width of the GaAs-related peak in Figure 1, equal to 0.4 T, agrees well with data in [28], [29].

In principle, as it is shown in [23], it is possible to obtain a PC signal also from undoped CdTe buffer layer, due to transitions in native donors, but this signal is much weaker than that of GaAs – related and can be neglected in current considerations.

The PC spectra in Figure 1 show another feature – a peak centered at about 2.2 T in all spectra, marked with a dashed line A. The signal rises abruptly at about 1.5 T, reaches the maximum at 2.2 T and vanishes at about 4.5 T. The half-width of the peak A is equal to about 0.8 T, and is much larger than that of 1s–2p^+^ transition in SI-GaAs. The peak A exhibits a nonsymmetric shape which is best visible in the lowest spectrum in Figure 1. It was verified that the peak A exhibits neither Gaussian nor Lorentzian shape. It looks rather like a superposition of many lines than a single spectral line. The most important observaton is sumarized in the inset to Figure 1 which shows that the position of the peak A does not change with FIR photon energy [38].

**Figure 2:**
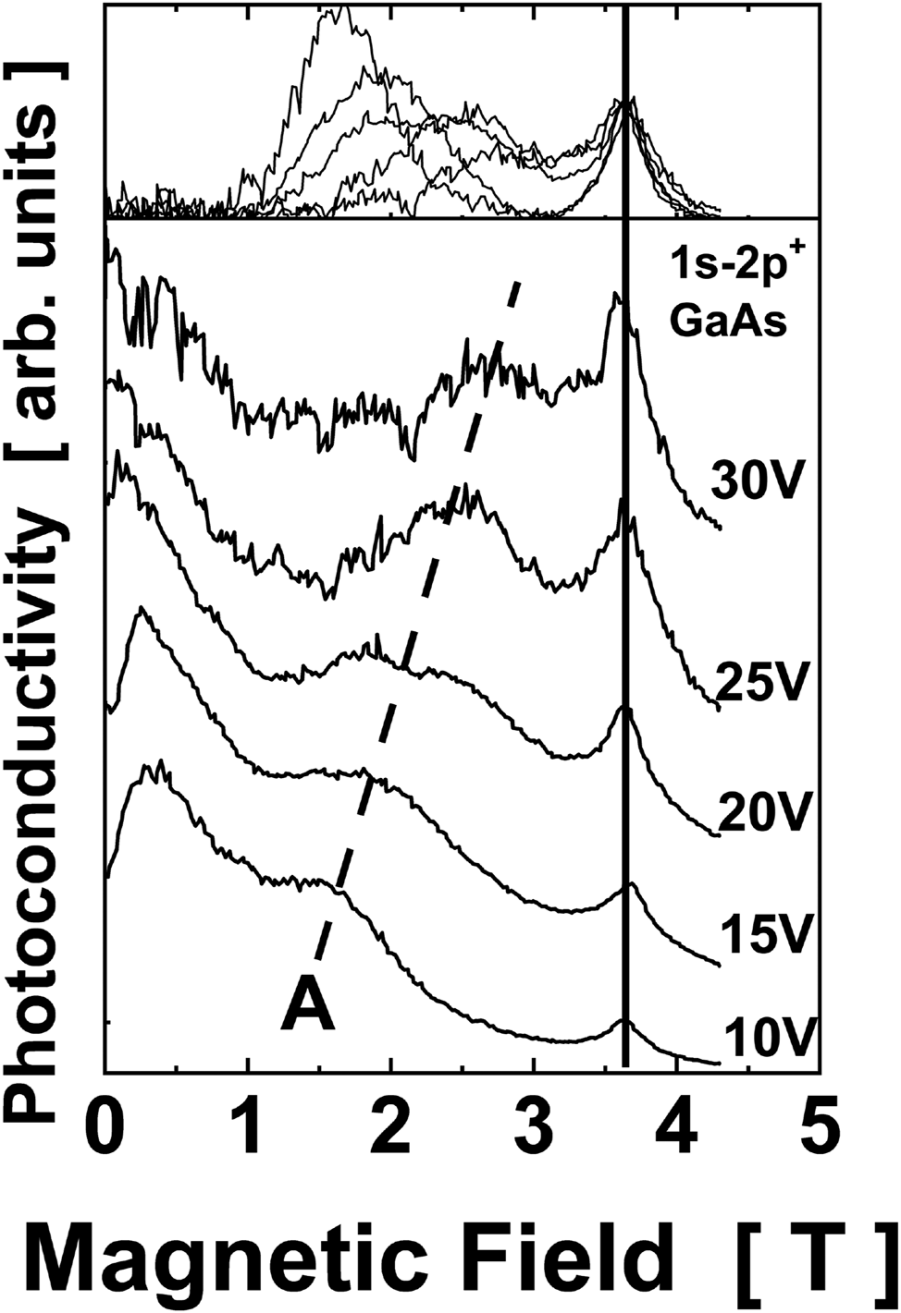
Evolution of the PC spectra as a function of applied voltage across the sample at a constant FIR photon energy *hν* = 10.4 meV at *T* = 4.2 K. The dashed line is the guide to the eye showing the position of the peak A. The spectra are labeled by the applied voltage *U* and are shifted vertically for clarity. The vertical solid line shows the position of 1s–2p^+^ transition in SI-GaAs. The inset shows the spectra after subtracting the background (assumed to be a smooth line connecting the low- *B* and high- *B* regions of the spectra) and normalization to the amplitude of 1s–2p^+^ GaAs peak.

Another unusual feature of the peak A is presented in Figure 2. The position of the peak A depends on the in-plane electric bias *U*: an increase of the bias shifts the peak towards higher magnetic fields. Let us note that the “standard” GaAs donor – related peak does not change the position with *U*.

The behavior of the peak A and that due to 1s–2p^+^ transition in GaAs shown in Figures 1 and 2 was observed in the whole range of the bias voltages (10 < *U* < 30 V) and FIR photon energies (2.2 meV < *hν* < 12.8 meV) applied.

According to the model presented below, we propose that the line A is a mixture of contributions from donors positioned in different places of the quantum well. The line A extends over a certain range of magnetic field (from about 1.5 T to 3.5T in Figure 1) but we do not expect an identical shape of the line A at different photon energy. Also, the shape of the line A in Figure 2 depends on the bias voltage preserving an overall shift to higher *B* with increasing bias but without strictly preserved shape. For these reasons, the inset to Figure 1 shows an approximate position of the middle of the range of *B* when the line A resides.

The experiment described in the present work demands the extreme care which is necessary to obtain reproducible results, defined as registering the same shape and position (in magnetic field) of the line A. It was verified that the line A was sensitive to the speed of cooling, intensity and photon energy of VIS illumination, mechanical stability of the insert and bias voltage. To obtain repeatable results, all these parameters had to be strictly controlled. In the case of an uncontrolled change of experimental conditions, it was necessary to heat the sample up to room temperature and repeat the experiment.

## 3 The model

The interpretation of experimental data is based on an essential assumption that the peak A is caused by intra-shallow impurity transitions, i.e., shallow iodine donors. This comes from the fact that with the energy of FIR photons used and the intensity of the magnetic field applied, one could – potentially – expect in that kind of experiment either shallow-impurity transitions or the cyclotron and magnetoplasmon resonances. The cyclotron resonance can be excluded because of a negligibly small concentration of electrons in the CB which appear there only as a result of the VIS illumination and independence of the position of the peak A on the photon energy. In turn, excitation of magnetoplasmons in two dimensions requires either a specially prepared periodic structure or, at least, a mesa necessary to quantize the wave vector of a plasmon [39]. Since we studied unprocessed samples, this possibility must also be excluded.

To construct a model explaining experimental observations, we consider a few factors which influence the PC in the magnetic field in the samples studied: the energy of intra-shallow donor transitions in quantum wells, a corresponding matrix element of these transitions and a magnetic-field-induced electron localization in fluctuations of the electrostatic potential.

### 3.1 Transition energy

In order to find electronic states of a shallow donor in a QW, the Schrödinger equation with the following Hamiltonian should be solved [2]:

(1) $$\hat{H}=\frac{p_{z}^{2}}{2m^{*}}+\frac{p_{x}^{2}+p_{y}^{2}}{2m^{*}}+V_{QW}+V_{C},$$

where *V*_*QW*_ is the potential of the QW and

(2) $$V_{C}=-\frac{e^{2}}{\kappa \sqrt{\rho^{2}+{\left(z-z_{i}\right)}^{2}}}$$

is the Coulomb potential of the shallow donor; m* is the electron effective mass, *κ* is the dielectric constant of the material of the QW, *ρ* is a projection of the vector of electron position onto the plane of the QW, and *z*_*i*_ is a position of the donor on the *z*-axis (assumed to be the direction of growth).

A solution to this problem gives energy levels of an isolated Coulomb center in a two-dimensional environment. Two features originating from such calculations are important in the case of our model:

(a) The energy of donor states depends on the width *L* of the QW. For *L* decreasing from the infinity to about $a_{B}^{*}$ , the binding energy *E*_*b*_ increases from the value of 1Ry* to a certain value between 1Ry* and 4Ry*. A further decrease of *L* leads to the decrease *E*_*b*_. This behavior is characteristic for any QW of a finite depth. In the case of an infinite barrier and *L* → 0, one gets the highest *E*_*b*_ = 4Ry*.
(b) The energy of donor states depends on the distance of a donor to the center of the QW. Let us underline that we consider here donors within the QW and these located in the barriers, which corresponds to a uniform doping of the structure. In the latter case, a donor center (positively charged) in the barrier bounds an electron which is in the QW.

**Figure 3:**
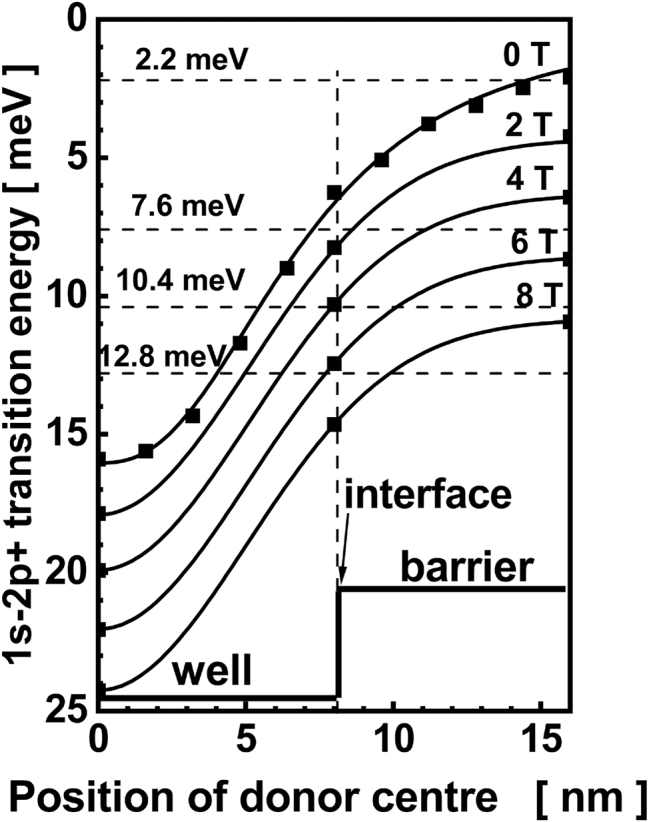
Dependence of the energy of 1s–2p^+^ transition in a 16 nm-thick and 240 meV-deep CdTe QW well as a function of the donor position in the structure. Squares – calculated energies after [2], lines – guide to the eye showing this dependence at different magnetic fields.

Figure 3 shows the results of calculations done on the basis of the above Hamiltonian in the case of the presence of the external magnetic field [15]. The transition energy between the ground state and the first excited state of a shallow donor for parameters corresponding to the QW considered in this paper is shown as a function of the donor position along the *z*-axis at different values of the magnetic field. The coordinate *z* = 0 corresponds to the middle of the QW, the values *z* > 8 nm correspond to the barrier region, what is schematically marked in the lower part of Figure 3. At zero magnetic field, the absorption due to 1s–2p^+^ transitions is possible for energies between about 6 and 16 meV for donors located in the QW (*z* < 8 nm). Since donors are located also in the barriers, the lower energy limit of possible transition is shifted down to about 2 meV.

**Figure 4:**
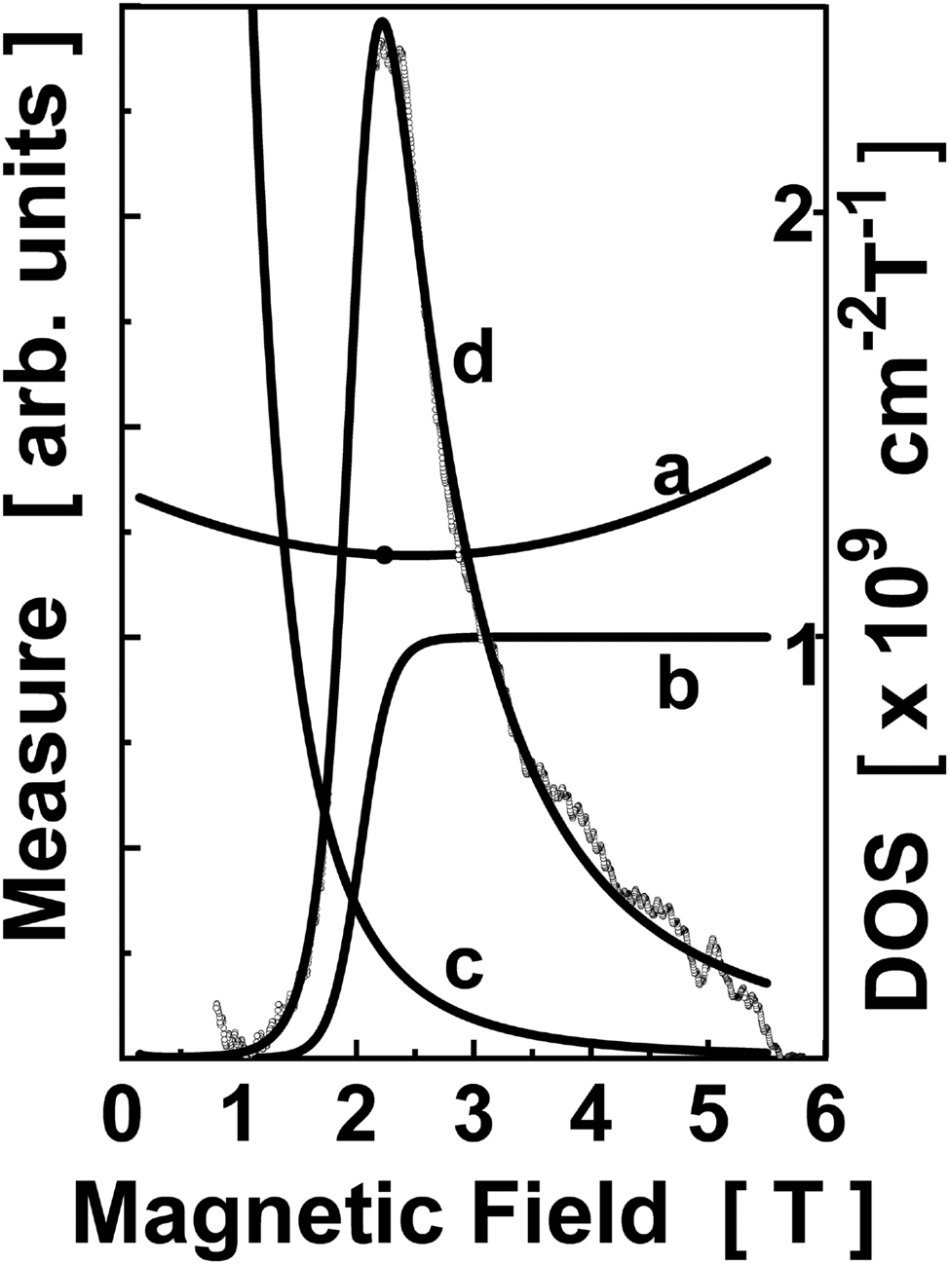
The peak A measured at *hν* = 12.8 meV (squares) compared to a theoretical fit (line). The data from Figure 1 was used (with the background subtracted). (a) Calculated density of donors states which can absorb photons of the energy *hν* = 12.8 meV (right scale): (b) qualitatively: the probability of population of the donor ground state due to localization induced by external magnetic field; (c) qualitatively: a combination of factors responsible for changes in the PC (see the text for a discussion); (d) superposition of (a), (b) and (c).

To discuss the FIR absorption at a non-zero magnetic field, let us concentrate on the photon energy equal to 12.8 meV (a FIR laser line of 118.8 μm). As can be concluded from Figure 3, sweeping the magnetic field up to 6 T one can observe absorption due to a resonant 1s–2p^+^ transtion on donors located at about 3.5 nm from the center of the QW (at *B* = 0) to these located next to the interface (at *B* = 6 T). In other words, one can say that at each *B*, a FIR photon “chooses” donors with energy levels appropriately split. One can calculate the density of these “appropriate” donor states (DOS). To this aim, at given *B*, the DOS as a function of donor position was determined using curves presented in Figure 3 and a constant sheet density of donors along the *z* axis. The calculations were done for 20 values of *B* between 0 and 5 T. The result of such a procedure is shown in Figure 4, curve *a* (similar curves were obtained for other photon energies).

The experimental curve (the line A at 12.8 meV) presented in Figure 4 was obtained from a corresponding curve in Figure 1 by subtraction of a linear background and by subtracting the GaAs-related peak. We assumed that the latter one is symmetrical and the high-B tail can be used to reproduce the low-B part.

This part of the model explains why it is possible to observe resonant 1s–2p^+^ transitions at a constant photon energy in a broad range of magnetic field – this is a result of a uniform doping of the QW and barriers. This does not explain other observations: why the position of the peak A does not depend on the photon energy but is very sensitive to an in-plane electric field. To continue, we consider next elements of the model. i.e., influence of fluctuations of the electrostatic potential and a magnetic-field induced electron localization.

### 3.2 A fluctuation pattern and localization

Fluctuations of the electrostatic potential result from a random distribution of charged centers. Let us recall that an extremely high mobility of a two-dimensional electron gas in GaAs-based heterostructures (and many other two-dimensional systems) results from separation of electrons in the well from their parent donor centers and a very small mismatch of the lattice constant of the well and barriers. Both these factors reduce electron scattering due to disorder. In the case of samples studied in this work, donors are introduced in the well and the barriers, which means that ionized centers are just in the very proximity to donors in which intra-center transitions occur. Next, a low concentration of free electrons, which appear as a result of VIS illumination only, imply a weak screening. Thus, one can expect a much higher amplitude of fluctuations than in QWs with doping layers separated from the well (samples with modulation – doped barriers) or QWs with a high concentration of mobile electrons.

As it was mentioned above, the position of the peak A in magnetic field depends on detailes of the cooling and VIS illumination of the sample. Such a sensitivity should be expected if the pattern of fluctuations of the electrostatic potential plays an important role in generation of the PC because different experimental conditions can lead to different patterns.

Simple estimations, based on statistical considerations, lead to a conlcusion that a random distribution of charged centers with a given concentration is a source of fluctuations which are characterized by a typical amplitude *V*_*fl*_ and a spatial extension *R*_*fl*_ [40]. Existence of a certain *R*_*fl*_ allows us to explain an increase of PC at certain *B* by noticing that when *B* grows electrons circle on a smaller and smaller cyclotron orbit, which increases their localization. If the radius of the cyclotron orbit *R*_*CR*_ is equal to about *R*_*fl*_ or is smaller, a probability of staying within a fluctuation of the height *V*_*fl*_ increases. In turn, the probability of capture of a localized electron by an ionized impurity also increases and results in a growth of the PC.

Thus we propose that an increase of the FIR PC at a certain magnetic field is due to a magnetic-field-induced localization of electrons in the CB of a disordered system which is followed by an increased population of neutral donors. In this picture, an increase of the PC should occur at given *B*, with no relation to the energy of incident photons. The magnetic-field induced localization occurs in the whole volume of the QW and – according to the way in which the sample is doped – creates a concentration of occupied donors with a broad distribution of transition energy, as explained above.

In other words, the magnetic – field induced localization is a trigger of the PC. This trigger – being defined by a relation between *R*_*fl*_ and *R*_*CR*_ only – occurs at given *B* which is independent on the photon energy.

The next observation to be explained is a decrease of the PC signal at high *B*. First of all, let us notice that the PC signal is generated due to resonant transitions within donors distributed in the quantum wells and the observed line A carries contributions from many impurities. Inspection of Figure 3 shows that at given energy of photons, the range of magnetic field in which the signal should be observed is limited. From this perspective, spectra shown in Figure 1 should be considered as composed of a background which starts at zero magnetic field and monotonically tends to zero at higher *B* and resonant structures which are superimposed on the background. One of them is a peak related to donors in GaAs substrate, the other is the line A. The background itself was not analyzed in the present paper.

Nevertheless, we would like to point at a few factors which can be responsible for a decrease of the PC signal at high *B*. First, the PC is a result of an optical transition accompanied by transport of excited electrons. Thus, a decrease of conductivity when the magnetic field grows reduces the PC. In a standard situation defined by a constant electron concentration, the conductivity decreases as ${\left(1+\mu^{2}B^{2}\right)}^{-1}$ which gives rather a slow decrease if the mobility *μ* is small, as it is expected in our case. However, in a disordered material, a freeze-out of electrons (i.e., their localization induced by the magnetic field) occurs due to growing density of localized states in the tail of the conduction band and concentration of electrons above the mobility edge can be strongly reduced [41].

Second, it is a decreasing value of the matrix element of the electric dipole transition between the ground and excited states of a shallow donor. In the process considered, it is the absorption of photons which finally leads to a photocurrent. Although a shrinkage of the wave functions in magnetic field leads to a decrease of the matrix element of the transition, the coefficient of absorption is proportional to the resonant frequency of the transition (see Eq. (5) in [42]) and this dependence can, at least partially, compensate a decrease of the materix element.

The third factor is related to the symmetry of the wave function of the ground and excited states of the donor. For a two-dimensional case, the symmetry of donor wavefunctions depends on the donor position with respect to the QW center [2]. The symmetry of the donor states at the center of the QW is close to the spherical one if the QW is much larger than the Bohr radius, as it is in the present case. For donors located at the interface, the ground state wavefunction becomes similar a p-like state since the wavefunction penetration into the barrier is exponentially weak and the symmetry of a *p*-state resembles a *d*-like state [42]. As one can conclude from Figure 3, when the magnetic field grows, the donors which are excited with radiation of a given photon energy are closer and closer to the interface. In general, an evolution of the matirx element with position of the donor was observed by a numerical analysis [12], [42], however, these calculations do not show a drastic reduction of the intensity of transitions for donors situated at the interface.

Thus, we conclude that there are a few factors which influence the tail of the line A at high magnetic fields and a detailed indication of their relative contributions is left for further analysis. For this reason, we qualitatively only present a “fitting” of the experimental curve in Figure 4 with lines which mimick expected combined influence of different factors.

## 4 Discussion

To summarize, we propose that the peak A is a superposition of contributions from 1s–2p^+^ transitions on donors positioned at different locations in the QW structure and influenced by a fluctuating electrostatic potential. Donors are mainly populated with the VIS illumination. The magnetic field leads to localization of electrons in fluctuations of the electrostatic potential once the cyclotron orbit fits into a characteristic extension of a fluctuation of a typical spacial extension. This increases the probability of occupation of shallow donors and leads to an increase of the PC. A decrease of the PC with *B* is expected to result from combined influence of a few factors which are discussed above.

Let us stress that the peak A is not a spectral line in a typical sense but rather a composition of single lines which can be attributed to donors spread over the barriers and the QW. The shape of the structure A is defined by the factors indicated in Figure 4 and is expected to be sensitive, in particular, to details of a donor distribution in the sample and details of the cooling procedure and parameters of the VIS illumination.

The proposed model easily explains a shift of the position of the peak A to a higher *B* when the in-plane electric field is increased. In fact, under the action of the bias voltage, fluctuations get shallower in the energy and smaller in extension (this effect resembles changes of the shape of a Coulomb potential when it is superimposed with a linear one). Then, a stronger magnetic field is required to achieve the condition *R*_*fl*_ ∼ *R*_*CR*_. We have to admit, however, that there is no simple relation between the bias voltage and the electric field within the sample. Experiments show that in the case of high-resistivity CdTe, both bulk and MBE grown, the applied bias drops practically immediately within a short distance adjacent to the positively polarized current contact [43]. Specifically, in an MBE-grown sample which was an undoped 3 μm-thick CdTe layer, as much as about 75 % of the applied voltage droped near this contact. This fact makes a recalculation of the bias voltage to the electric field in the sample very difficult.

We expect that features analogous to the peak A could be observed in other types of uniformly doped high-resistivity QWs where a high degree of compensation leads to the appearance of a fluctuating pattern of the electrostatic potential. Optimization of parameters of such structures (the doping level, the barriers composition) could lead to fabrication of a sensitive and external voltage-tunable detector of FIR radiation.
